# Vasoactive Soluble Endoglin: A Novel Biomarker Indicative of Reperfusion after Cerebral Large-Vessel Occlusion

**DOI:** 10.3390/cells12020288

**Published:** 2023-01-11

**Authors:** Axel Haarmann, Christoph Vollmuth, Alexander M. Kollikowski, Peter U. Heuschmann, Mirko Pham, Guido Stoll, Hermann Neugebauer, Michael K. Schuhmann

**Affiliations:** 1 Department of Neurology, University of Würzburg, 97080 Würzburg, Germany; 2 Department of Neuroradiology, University of Würzburg, 97080 Würzburg, Germany; 3 Institute of Clinical Epidemiology and Biometry, University of Würzburg, 97080 Würzburg, Germany; 4 Clinical Trial Center, University Hospital Würzburg, 97080 Würzburg, Germany

**Keywords:** endoglin, brain endothelium, stroke, shedding, mechanical thrombectomy, hypoxia, reperfusion injury, biomarker

## Abstract

Now that mechanical thrombectomy has substantially improved outcomes after large-vessel occlusion stroke in up to every second patient, futile reperfusion wherein successful recanalization is not followed by a favorable outcome is moving into focus. Unfortunately, blood-based biomarkers, which identify critical stages of hemodynamically compromised yet reperfused tissue, are lacking. We recently reported that hypoxia induces the expression of endoglin, a TGF-β co-receptor, in human brain endothelium in vitro. Subsequent reoxygenation resulted in shedding. Our cell model suggests that soluble endoglin compromises the brain endothelial barrier function. To evaluate soluble endoglin as a potential biomarker of reperfusion (-injury) we analyzed its concentration in 148 blood samples of patients with acute stroke due to large-vessel occlusion. In line with our in vitro data, systemic soluble endoglin concentrations were significantly higher in patients with successful recanalization, whereas hypoxia alone did not induce local endoglin shedding, as analyzed by intra-arterial samples from hypoxic vasculature. In patients with reperfusion, higher concentrations of soluble endoglin additionally indicated larger infarct volumes at admission. In summary, we give translational evidence that the sequence of hypoxia and subsequent reoxygenation triggers the release of vasoactive soluble endoglin in large-vessel occlusion stroke and can serve as a biomarker for severe ischemia with ensuing recanalization/reperfusion.

## 1. Introduction

Although mechanical thrombectomy (MT) has improved the recanalization rates and outcomes in large-vessel occlusion (LVO) stroke, many patients do not experience a favorable outcome despite timely and successful recanalization. Recanalization of basal cerebral arteries does not necessarily lead to reperfusion of the adjacent microvascular beds where complex endothelium–leukocyte–platelet interactions are likely to contribute to focal no-reflow [1]. Accordingly, there is increasing interest in identifying blood-based biomarkers which reflect critical stages of infarct development including reperfusion in the microcirculation [2]. Candidate biomarkers are often derived from findings in experimental stroke models. We recently reported a mechanism in which the sequence of hypoxia followed by reoxygenation leads to the release of membrane-bound endoglin (ENG) in vitro. ENG, as a coreceptor of TGF-β, is an important modulator of vascular homeostasis: ENG deficiency is embryonically lethal, and heterozygous mutations within the ENG gene result in hereditary hemorrhagic telangiectasia, a vascular disease with arteriovenous malformations and bleeding complications. Membrane-bound ENG can be shed by matrix metalloproteinase-12 and -14, the latter of which is upregulated under hypoxia. Soluble ENG (sENG), in turn, caused a dose-dependent increase in paracellular permeability and downregulation of VE-cadherin in our cell model [3]. In summary, we hypothesized that sENG is extensively shed upon hypoxia and subsequent reoxygenation and is a pro-inflammatory mediator at the human brain endothelium. Thus, in acute ischemic stroke treated with MT, reperfusion of the microcirculation potentially triggers the release of vasoactive molecules that add to endothelial dysfunction and might exacerbate ischemia/reperfusion (I/R) injury, partly driven by thrombo-inflammation [1]. At present, the contribution of soluble endothelial mediators such as ENG, where shedding is dependent on hypoxia and subsequent reperfusion, is not clear. We analyzed sENG concentrations in plasma samples of ischemic stroke patients with LVO in the anterior circulation undergoing MT. By comparing specimens collected under hypoxia by intra-arterial aspiration distal to occluding thrombi and intravenously after various degrees of reperfusion, we validated our in vitro data of sENG as a biomarker for microvascular reperfusion and I/R injury in stroke patients. 

## 2. Materials and Methods

### 2.1. Patients, Blood Samples and Neuroradiological Scoring

We identified 148 patients, enrolled into a single-center prospective study (DRKS00022064), who were admitted to the University Hospital Würzburg between June 2020 and February 2022 with acute stroke due to cerebral LVO of the anterior circulation. Intra-arterial blood acquisition was performed during MT in the internal carotid artery (as an intra-individual control) and distal of the occluding thrombi immediately after penetration but prior to recanalization. Therefore, the microwire and microcatheter were navigated past the embolus into the midinsular M2 segment located within the center of the ischemic circulation, exactly as formerly described [4]. Patients undergoing this procedure had to meet clinical and imaging inclusion/exclusion criteria. In brief, these were: first-ever ischemic stroke with a deficit corresponding to a National Institute of Health stroke scale (NIHSS) of at least 6, exclusion of hemorrhage or large infarct volume (<5 in the Alberta stroke program early CT- (ASPECT-) score), exclusion of multifocal LVO, vessel sub-occlusion or LVO in association with either ≥50% common carotid artery or cervical ICA stenosis or ICA dissection, the requirement of percutaneous transluminal angioplasty, stent implantation or intra-procedural platelet inhibitor use. For more details, see [4]. Citrate plasma was snap frozen and stored at −80 °C until analysis. The intravenous blood samples were collected at 10:00 a.m. the day after MT, resulting in a mean interval between the procedure and acquisition of 18.2 h (±8.4). EDTA plasma was snap frozen and stored at −80 °C until analysis. 

This study was approved by the local ethics committee (approval # 135/17 and # 05/20-am). All the patients or their legal representatives provided written informed consent. The ASPECT and eTICI scores were assessed by experienced neuroradiologists.

### 2.2. Enzyme-Linked Immunosorbent Assay

For the analysis of the sENG concentration, patient samples were diluted 1:3 and subjected to the human endoglin Quantikine ELISA Kit (#DNDG00) by R&D Systems (Minneapolis, MN, USA) according to the instructions in the product insert and measured with a TECAN (Maennedorf, Switzerland) infinite 200 PRO ELISA reader. 

### 2.3. Statistics

Statistical analysis was performed with GraphPad PRISM Version 9.4.0 (La Jolla, CA, USA). For comparison of multiple groups, we performed the non-parametric Kruskal–Wallis test followed by Dunn’s post-test. For comparison of two groups, we performed a T-test using the nonparametric Kolmogorov–Smirnov test (* *p* < 0.05; ** *p* < 0.01). Correlation was examined using Spearman correlation coefficient.

## 3. Results

### 3.1. Elevated Systemic sENG Concentrations Indicate Successful Reperfusion and Reflect Infarct Volume

In total, we included 148 plasma samples of patients with LVO in the anterior circulation in our analysis, of which 129 patients (87%) were treated with MT. A total of 40.5% of the patients received the thrombolytic agent recombinant tissue plasminogen activator (rt-PA). The mean age was 76.2 (74.1–78.2) years. A synopsis of the patients’ characteristics is shown in Table 1. 

**Figure 1:**
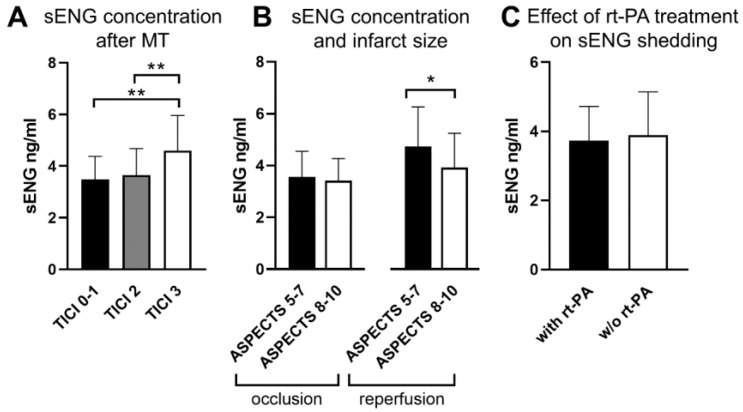
Concentration of sENG in venous plasma samples of stroke patients with LVO in the anterior circulation. Blood samples were drawn 18.2 (±8.4) hours after MT and subjected to ELISA. In the 129 patients undergoing MT, sENG levels were significantly higher after complete (TICI 3) recanalization (TICI 0–1: *n* = 10, 20% received rt-PA; TICI 2: *n* = 94, 38.5% received rt-PA; TICI 3: *n* = 25, and 60% received rt-PA; there was no significant difference in the ASPECT scores between these groups) (**A**). In patients with successful recanalization, larger infarct volumes, as reflected by a lower ASPECT score at admission, resulted in increased sENG concentrations (148 patients including 19 without MT) (**B**). Application of rt-PA alone or in addition to MT did not influence shedding of sENG (black bar reflects 60 patients receiving rt-PA (80% with MT); white bar shows 88 patients without rt-PA treatment (92% with MT) (**C**)). Graphs depict means and standard deviation. Non-parametric Kruskal–Wallis test followed by Dunn’s post-test in A, T-test by the nonparametric Kolmogorov–Smirnov test in B and C; (* *p* < 0.05, ** *p* < 0.01).

As recently reported, membrane-bound ENG is upregulated upon hypoxia and shed after subsequent reoxygenation by human brain endothelial cells [3]. Thus, we sought to determine whether the degree of recanalization after MT would affect the concentration of sENG. In fact, patients with complete reperfusion corresponding to grade 3 in the thrombolysis in cerebral infarction- (TICI-) grading system showed a significant increase in sENG concentrations compared to missing or only partial recanalization (Figure 1A). We then hypothesized that the size of the affected endothelial surface area (stroke volume) would influence sENG concentrations. In clinical practice, infarct size in the middle cerebral artery (MCA) territory is estimated using the ASPECT score, which is a semi-quantitative measure of infarcted hypoxic tissue [5]. As expected, patients with large (ASPECTS 5-7) infarcts at admission and subsequent successful reperfusion had significantly higher sENG concentrations compared to patients without reperfusion (3.7 [3.1–4.2] vs. 4.7 [3.8–5.6] ng/mL; *p* = 0.013). Correspondingly, without recanalization, there was no difference in the sENG concentrations of patients with large (ASPECTS 5–7) or small (ASPECTS 8–10) infarcts at admission. In patients with successful recanalization, however, more severe tissue damage was associated with an increase in sENG (Figure 1B). These data underline that the release of sENG is dependent on reperfusion after hypoxia and infarct size at admission.

To validate that, also locally in the ischemic vasculature, sENG shedding did not occur prior to reoxygenation, we analyzed 31 acute stroke patients with arterial samples of microcatheter aspiration from the occluded vessel area (before recanalization), as previously described [4]. Intra-arterial aspiration of carotid blood samples served as an intra-individual control (Table 1). We found no difference in sENG concentration between ischemic and control samples, indicating that hypoxia alone is not sufficient to induce sENG release in the infarcted area, underscoring the importance of subsequent reperfusion in cerebral sENG shedding in vivo.

### 3.2. Recombinant Tissue Plasminogen Activator Treatment Does Not Change sENG Release in LVO Stroke Patients

We recently described that rt-PA reaches the microcirculation distal of LVO independent of reperfusion and that it can cause an inflamed phenotype in murine brain endothelial cells [6,7]. Therefore, we hypothesized that rt-PA treatment might alter sENG shedding in acute stroke patients independent of MT. Of 148 patients with LVO, 60 (40.5%) received rt-PA treatment. This did not change the systemic sENG levels (Figure 1C). 

### 3.3. Concentrations of sENG Positively Correlate with NIHSS after 72 h 

**Figure 2:**
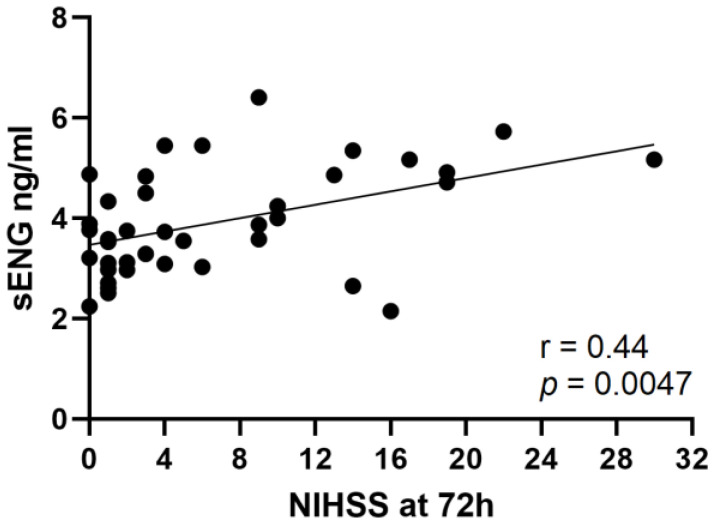
Positive correlation of sENG concentrations with the NIHSS 72 h after stroke onset in patients with partial or complete (corresponding to TICI 2C or TICI 3) recanalization. Spearman correlation coefficient (*n* = 39, r = 0.44, *p* = 0.0047).

After successful recanalization, higher sENG concentrations are indicative of larger infarct volumes. Thus, in these patients, sENG concentrations were correlated with the symptom severity (Figure 2). As thrombectomy is usually performed under general anesthesia in our center, neurological assessment is impaired in the early phase. Therefore, we assessed the NIHSS 72 h after stroke onset. Spearman correlation analysis showed a positive relationship between sENG concentrations with the NIHSS after 72 h (*p* = 0.0047, r = 0.44).

## 4. Discussion

The identification of stage-specific blood biomarkers is a rapidly evolving field of stroke research. In LVO stroke, successful removal of the occluding thrombi inevitably leads to the sequence of hypoxia followed by reoxygenation in the ischemic (micro-) vascular bed. Applying an in vitro model of cerebral I/R injury, we recently demonstrated that membrane-bound endoglin is upregulated in brain endothelial cells under hypoxia and cleaved (shed) upon reoxygenation. Soluble endoglin, as a vasoactive mediator, in turn induced endothelial barrier dysfunction [3,8].

As a principal finding we here show that ENG is released into the human circulation during acute stroke, a process which is largely dependent on reperfusion. Hypoxia alone did not increase sENG concentrations, neither locally in the ischemic vasculature, nor systemically. Only after sufficient reperfusion, we were able to show a release of ENG that indicated recanalization accordingly. Of note, larger infarct volumes at admission were associated with higher sENG plasma concentrations. This is in good agreement with our hypothesis that hypoxic activation of brain endothelial cells, as a potent source of sENG, is a prerequisite for ENG shedding upon reperfusion and argues for sENG as a marker for the reperfused microcirculation.

Shedding of membrane proteins is a common mechanism in endothelial inflammation including brain endothelial cells [9]. Cleavage of receptors and molecules does not only modulate intracellular pathways but can also induce outside-in signaling in an autocrine fashion, as reported for sVCAM-1 impairing human brain endothelial barrier integrity [10]. Shedding of molecules is also likely to directly influence endothelial/leukocyte- and endothelial/platelet-interactions, which have recently gained increasing attention as intravascular players of human and experimental stroke [1,11,12]. Besides these mechanisms that might potentially add to reperfusion injury, such molecules, could also be used as early biomarkers for reperfusion and infarct size.

## 5. Conclusions

In summary, we show that, in acute stroke patients with LVO, the sequence of hypoxia and subsequent reoxygenation induces shedding of soluble endoglin. Soluble ENG, in turn, can serve as a biomarker for severe ischemia with ensuing recanalization/reperfusion. Better characterization of the “sheddome” of human brain endothelium after hypoxia and subsequent reperfusion is challenging but would be very valuable in the context of I/R injury.

## Figures and Tables

**Table 1:** Demographic, clinical and laboratory characteristics of the patients (*n* = 148).

Variable	%	Mean	95% CI	min.–max.	*n*
Age (y)		76.2	74.1–78.2	22–99	148
Female sex	54.7				81
Subjected to MT	87.0				129
Received i.v. rt-PA	40.5				60
ASPECTS		7.4	7.1–7.6	1–10	148
sENG concentration i.v. (ng/mL)		3.77	3.6–3.9	1.9–8.0	148
sENG concentration i.a. cerebral (ng/mL)		2.2	1.9–2.5	0.9–4.1	31
sENG concentration i.a. systemic (ng/mL)		2.1	1.8–2.4	0.6–4.1	31

## Data Availability

The datasets used and/or analyzed during the current study are available from the corresponding author on reasonable request.

## Abbreviations

Large vessel occlusion (LVO), ischemia/reperfusion (I/R), endoglin (ENG), soluble endoglin (sENG), mechanical thrombectomy (MT), middle cerebral artery (MCA), thrombolysis in cerebral infarction- (TICI-), The Alberta stroke programme early CT score (ASPECTS).
